# Rebound Hyperkalemia After Potassium Repletion in Thyrotoxic Periodic Paralysis: A Case Report and Review of Management Implications

**DOI:** 10.7759/cureus.85551

**Published:** 2025-06-08

**Authors:** Daichi Matsui, Shin Mugikura, Takahiro Goshima, Noriyoshi Ishizuka, Naruhiro Jingushi, Norimichi Uenishi, Mitsunaga Iwata

**Affiliations:** 1 Department of Emergency and General Internal Medicine, Fujita Health University School of Medicine, Toyoake, JPN

**Keywords:** endocrinology, hypokalemia, propranolol, rebound hyperkalemia, thyrotoxic periodic paralysis

## Abstract

Thyrotoxic periodic paralysis (TPP) is a potentially life-threatening complication of hyperthyroidism. It is characterized by hypokalemia-induced muscle weakness that typically begins in the proximal lower limbs and may progress to paralysis of all four extremities and involvement of the respiratory muscles.

We present a case of a 37-year-old man with a history of TPP, presenting with acute muscle weakness and hypokalemia. The patient reported acute-onset bilateral lower extremity weakness from the previous day. Physical examination revealed normal deep tendon reflexes, but marked muscle weakness was observed in both lower limbs. Laboratory workup revealed severe hypokalemia at 2.1 mEq/L and thyrotoxicosis, while the electrocardiogram showed a prolonged QTc interval. The patient received thiamazole, potassium iodide, and propranolol for thyrotoxicosis and a total dose of 122 mEq of potassium repletion. His potassium level rose from 1.7 mEq/L to 5.6 mEq/L within six hours post-repletion cessation, highlighting the risk of rebound hyperkalemia and the importance of close monitoring.

This case underscores the danger of rebound hyperkalemia after aggressive potassium repletion in TPP and supports a cautious, stepwise correction strategy.

## Introduction

Thyrotoxic periodic paralysis (TPP) is a rare but potentially serious complication of hyperthyroidism, characterized by sudden-onset muscle weakness and hypokalemia. It is more commonly reported in Asian males [1]. TPP results from increased Na⁺/K⁺-ATPase activity triggered by excess thyroid hormones and enhanced β-adrenergic stimulation, which promotes intracellular potassium shift [2]. Precipitating factors include high carbohydrate intake, which raises insulin levels, and physical exertion, which increases catecholamine release, both of which stimulate Na⁺/K⁺-ATPase activity and exacerbate hypokalemia [3]. Clinically, TPP typically presents with acute proximal muscle weakness, especially in the lower limbs, and may progress to involve all four limbs and the respiratory muscles [4].

Severe hypokalemia is a medical emergency that can cause life-threatening cardiac arrhythmias. While potassium supplementation is generally required, overly aggressive correction may lead to rebound hyperkalemia, which poses a similar risk of fatal arrhythmias and respiratory arrest [5,6]. We report a case of TPP complicated by rebound hyperkalemia, emphasizing the importance of cautious potassium repletion in the management of this condition.

## Case presentation

**Figure 1:**
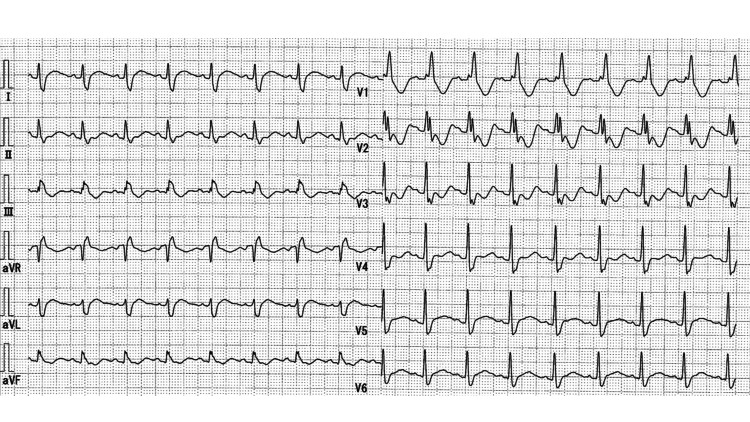
Initial electrocardiogram showing sinus tachycardia and QTc prolongation due to hypokalemia.

A 37-year-old man presented to our hospital with acute-onset bilateral lower extremity weakness that began the previous day. He had no past medical history, was not taking any medications, and denied alcohol use. On arrival, his vital signs were as follows: Glasgow Coma Scale score of 15 (E4V5M6), blood pressure of 150/105 mmHg, heart rate of 100 beats per minute, respiratory rate of 20 breaths per minute, oxygen saturation of 97% on room air, and body temperature of 36.2°C. Neurological examination revealed decreased strength in the bilateral iliopsoas muscles (manual muscle test score 3/5), while both patellar and Achilles tendon reflexes were preserved. Laboratory tests showed hypokalemia and hyperthyroidism (Table 1). Electrocardiography revealed sinus tachycardia with a markedly prolonged QTc interval of 0.564 seconds, consistent with hypokalemia-induced cardiac excitability (Figure 1). Based on the clinical findings, a diagnosis of TPP was made. The patient denied any recent high-carbohydrate meals or strenuous physical activity.

**Table 1:** The patient's laboratory investigations at presentation. TSH: thyroid-stimulating hormone; F-T4: free thyroxine; F-T3: free triiodothyronine; TRAb: thyrotropin receptor antibody.

Laboratory test	Patient's result	Reference range
Biochemistry		
C-reactive protein	0.11	<0.14 mg/dL
Creatine phosphokinase	796	59-248 IU/L
Creatinine	0.46	0.65-1.07 mg/dL
Sodium	142	138-145 mEq/L
Potassium	2.1	3.6-4.8 mEq/L
Chloride	109	101-108 mEq/L
Calcium	8.3	8.8-10.1 mg/dL
Phosphorus	2.2	2.7-4.6 mg/dL
Magnesium	1.7	1.8-2.4 mg/dL
Hormonal data		
TSH	0.006	0.500-4.800 μIU/mL
F-T4	2.91	0.83-1.77 ng/dL
F-T3	12.56	2.51-4.16 pg/mL
TRAb	7.7	<2.0 IU/L
Urinary biochemistry		
Potassium-to-creatinine ratio	10.7	<13 mEq/gCr

**Figure 2:**
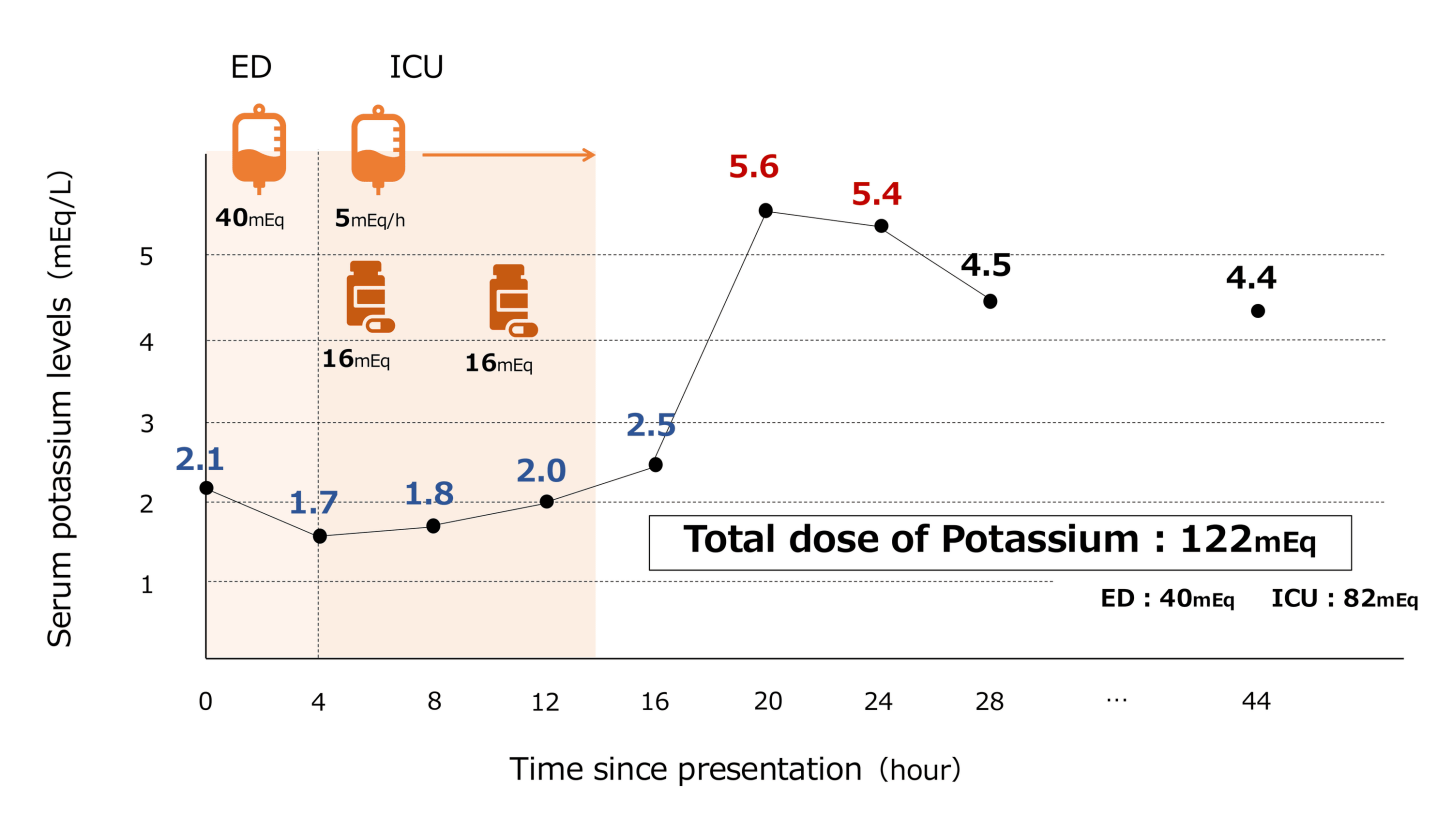
Clinical course of serum potassium levels and potassium repletion after presentation. ED: emergency department; ICU: intensive care unit.

**Figure 3:**
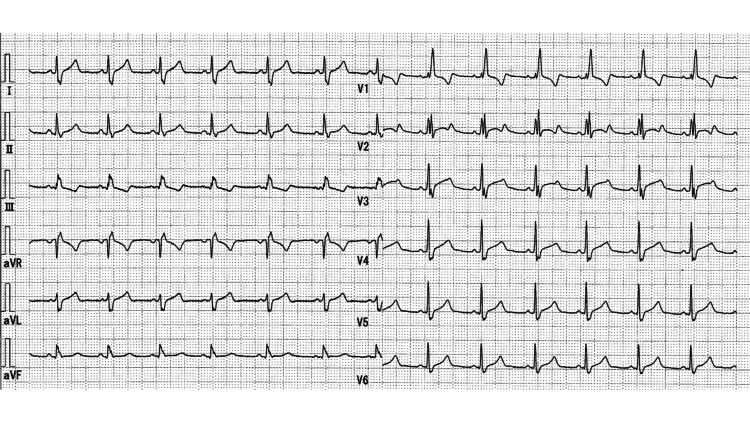
Electrocardiogram on hospital day two showing improvement in the QTc interval following treatment.

The post-admission course of serum potassium levels and potassium repletion is presented in Figure 2. In the emergency department, he received intravenous potassium chloride (KCl) at 40 mEq over four hours. However, his serum potassium level further declined to 1.7 mEq/L upon admission to the intensive care unit (ICU). Antithyroid treatment was initiated with oral thiamazole 10 mg/day, potassium iodide 50 mg/day, and propranolol 20 mg three times daily. Potassium repletion was continued using both oral (KCl 16 mEq per dose) and intravenous routes (KCl 5 mEq/hour; 20 mEq KCl in 30 mL saline over four hours via a central venous catheter). During the first 10 hours in the ICU, the patient received a total of 82 mEq of potassium (32 mEq orally and 50 mEq intravenously). Despite a still low potassium of 2.0 mEq/L, repletion was halted due to concern for rebound hyperkalemia, a known risk in TPP where intracellular redistribution, not deficit, drives hypokalemia. Two hours later, the level increased to 2.5 mEq/L, and rose further to 5.6 mEq/L after an additional four hours (six hours post-repletion cessation). No active intervention was required, as the potassium level gradually normalized to approximately 4.5 mEq/L. The next day, the patient’s electrocardiogram showed improvement, with a QTc interval shortened to 0.481 seconds (Figure 3). Muscle strength recovered, and he was transferred out of the ICU on hospital day two. He was discharged on day five with endocrinology follow-up and continuation of thiamazole and propranolol for definitive management of thyrotoxicosis.

## Discussion

TPP leads to hypokalemia as thyroid hormones increase the responsiveness of β_2_ receptors, activating Na^+^/K^+^ ATPase on the skeletal muscle membrane, which transports potassium into the intracellular space [2]. Factors such as elevated insulin levels from excessive carbohydrate intake and increased catecholamines from strenuous exercise can worsen the condition by further activating Na^+^/K^+^ ATPase [3]. However, a large prospective cohort study reported that a clear precipitating factor was identified in only 34% of TPP patients, indicating that only a small fraction has clear precipitating factors [4]. In this case, no specific event that could have triggered the onset was identified.

In TPP-induced hypokalemia, potassium repletion can shorten the recovery time to improve serum potassium levels; however, there is a reported risk of rebound hyperkalemia [5,6]. This risk arises because the cause of hypokalemia in TPP is not a deficiency but an intracellular shift of potassium. When this shift is reversed, potassium returns to the extracellular space. For example, a reported TPP case receiving 240 mEq of potassium resulted in rebound hyperkalemia of 10.1 mEq/L and death, illustrating the lethal potential of overcorrection [7]. Hypothermia therapy and insulin therapy can also cause hypokalemia due to intracellular potassium shifts. There have been case reports of rebound hyperkalemia occurring upon rewarming or after discontinuation of an insulin pump [8,9]. Clinicians should administer cautiously in cases of hypokalemia caused by intracellular shifts. A study demonstrated rebound hyperkalemia (K > 5.0 mEq/L) in approximately 40% of patients with TPP who received more than 90 mEq of potassium within 24 hours [10]. While no consensus exists, evidence suggests that limiting potassium repletion to ≤90 mEq in 24 hours may minimize risk. Further studies are warranted to define safe thresholds. In this case, the patient received a total dose of 122 mEq of potassium repletion.

On the other hand, propranolol, a non-selective β-blocker, is gaining attention as an alternative treatment to potassium repletion in TPP. Propranolol suppresses the intracellular shift of potassium by inhibiting the Na^+^/K^+^ ATPase pump activity, which helps prevent the decrease in serum potassium levels. Propranolol mitigates the intracellular potassium shift by β_2_ blockade, with some studies showing effective monotherapy that avoids rebound hyperkalemia seen with potassium alone [11,12]. In this case, propranolol was used; however, a large amount of potassium repletion was administered, which ultimately led to rebound hyperkalemia. In the future, for the safe treatment of hypokalemia in TPP, it is considered necessary to conduct further clinical trials to determine the optimal dosage of propranolol or explore combination therapies of propranolol and potassium repletion.

## Conclusions

TPP is a potentially life-threatening condition characterized by acute limb weakness and hypokalemia. While potassium repletion is essential for correcting hypokalemia, clinicians must remain vigilant about the risk of rebound hyperkalemia, particularly when intracellular potassium shifts reverse. Although propranolol is considered a promising adjunctive treatment that targets the underlying pathophysiology without inducing dangerous electrolyte disturbances, the risk of rebound hyperkalemia remains if potassium repletion is not carefully managed. Meticulous potassium correction and vigilant monitoring of serum potassium levels are crucial for optimizing patient outcomes. Further studies are needed to establish evidence-based guidelines for the optimal dosing of potassium and propranolol in the management of TPP to ensure safe and effective clinical outcomes.
